# Perception of global image contrast involves transparent spatial filtering and the integration and suppression of local contrasts (not RMS contrast)

**DOI:** 10.1098/rsos.170285

**Published:** 2017-09-06

**Authors:** Tim S. Meese, Daniel H. Baker, Robert J. Summers

**Affiliations:** 1 School of Life and Health Sciences, Aston University, Birmingham B4 7ET, UK; 2 Department of Psychology, University of York, York YO10 5DD, UK

**Keywords:** human vision, contrast summation, Battenbergs, contrast gain control, image processing, psychophysics

## Abstract

When adjusting the contrast setting on a television set, we experience a perceptual change in the global image contrast. But how is that statistic computed? We addressed this using a contrast-matching task for checkerboard configurations of micro-patterns in which the contrasts and spatial spreads of two interdigitated components were controlled independently. When the patterns differed greatly in contrast, the higher contrast determined the perceived global contrast. Crucially, however, low contrast additions of one pattern to intermediate contrasts of the other caused a paradoxical *reduction* in the perceived global contrast. None of the following metrics/models predicted this: max, linear sum, average, energy, root mean squared (RMS), Legge and Foley. However, a nonlinear gain control model, derived from contrast detection and discrimination experiments, incorporating wide-field summation and suppression, did predict the results with no free parameters, but only when spatial filtering was removed. We conclude that our model describes fundamental processes in human contrast vision (the pattern of results was the same for expert and naive observers), but that above threshold—when contrast pedestals are clearly visible—vision's spatial filtering characteristics become transparent, tending towards those of a delta function prior to spatial summation. The global contrast statistic from our model is as easily derived as the RMS contrast of an image, and since it more closely relates to human perception, we suggest it be used as an image contrast metric in practical applications.

## 1. Background

**Figure 1.**
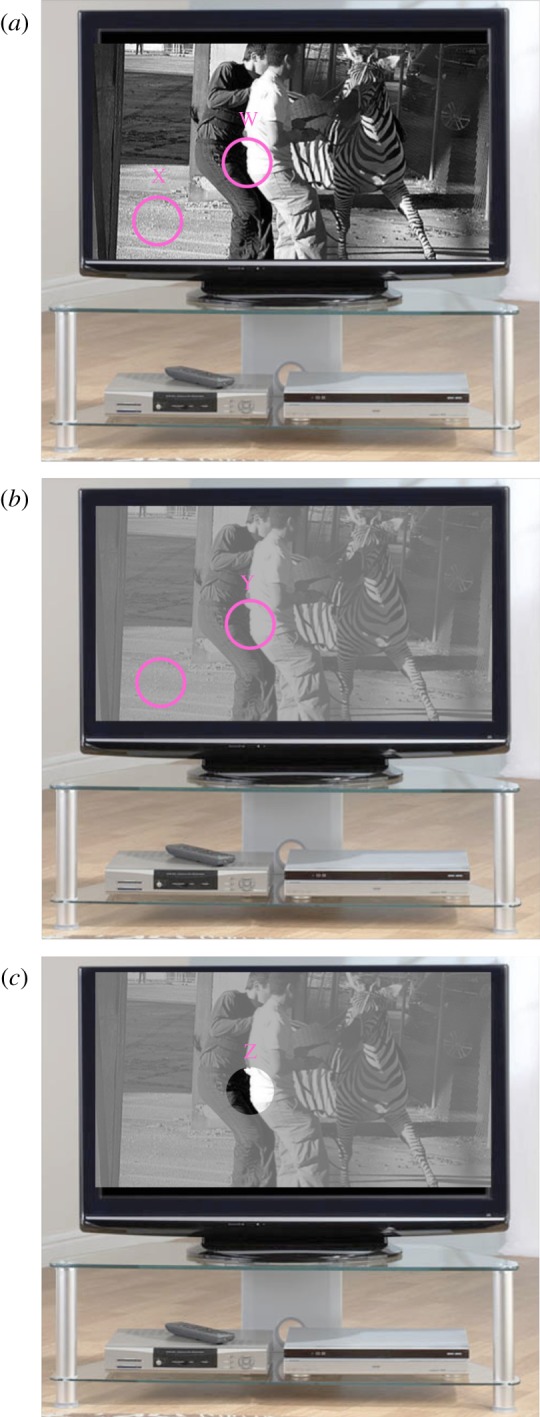
Black and white images can be judged in terms of both their local contrasts (W versus X in (*a*)) and their global contrast ((*a*) versus (*b*)). They can also be segmented: image (*c*) is the same as image (*b*), but with the small segment of W from image (*a*) superimposed (Z); the global contrast of (*c*) looks very much the same as (*b*), but with a local high contrast circular patch (Z). In spite of this local high contrast, it does not appear to have the same global image contrast as image (*a*), suggesting that perception of global contrast involves some form of contrast aggregation (and possibly, segmentation). The RMS and Michelson contrasts, respectively (see text for definitions), for the different images and regions are as follows: high contrast image (*a*) = 27% & 100%; low contrast image (*b*) = 8% & 29%; low contrast image with high contrast sub-region (*c*) = 10% & 100%; region W (& Z) = 44% & 100%; region X = 6% & 68%; region Y = 13% & 29%. Note that according to the Michelson contrast, image (*a*) and (*c*) are the same (100%), which does not accord with casual perception. Note also that the RMS contrast (a widely used metric for image contrast, applicable to both local and global measures) in region Y (in (*b*)) is higher than in region X (in (*a*)), indicating that this measure of local contrast should not be used to infer the global image contrast, since the overall contrast of image (*a*) looks higher than image (*b*). We present a more formal analysis of this general point in part 1 of the electronic supplementary material.

When we look at an image on a black and white television set, we might notice two things about its contrast. First, the local contrasts vary across the image. For example, in figure 1*a*, the local region W has a much higher contrast than the local region X. Second, in spite of these variations in local contrasts, we are able to make a judgement about the *global* image contrast. For example, the global contrast in figure 1*a* is clearly higher than that in figure 1*b*, even though there are regions in figure 1*b* (e.g. region Y) that have local contrasts which are higher than other regions in the high contrast image (e.g. region X) (see figure caption for further details.). In other words, when we adjust the contrast control on a television set, we change both local and global contrasts together, but the perception of overall contrast cannot be given by a simple local contrast measure, because local contrasts vary across the image [1,2]. Thus, the perception of global image contrast must involve some form of assessment across the entire image; how might that be done?

### 1.1. Global contrast metrics

While the perception of contrast has attracted a great deal of attention from the vision community, the issues of whether and, if so, how global contrast might be measured has received surprisingly little. One candidate measure is the Michelson contrast of the image, given by: *C*_Michelson_ = (*L*_max_ − *L*_min_)/(*L*_max_ + *L*_min_), where *L*_max_ and *L*_min_ are the maximum and minimum luminance measures across the entire image, respectively. This measure has the benefit of simplicity, but captures little of the variation in contrast levels across the image, being dependent on the luminance levels at just two locations. Thus, suppose that all of the other luminance levels were either increased to just below *L*_max_, or decreased to just above *L*_min_; these manipulations would not change the Michelson contrast of the image, but would have substantial effects on its perceived contrast. Another simple measure is to compute local contrasts across the image, and simply choose the largest. But this measure suffers from similar problems to those of the Michelson contrast. For example, the TV picture in figure 1*c* looks to be of much lower global contrast than that in figure 1*a*, even though it contains the same maximum high contrast region as that in figure 1*a* (W = Z); it seems that what is needed is some form of contrast aggregation. The simplest is the contrast sum: the sum of the local contrasts across the image. A related metric is that of contrast energy: the sum of the squares of the local contrasts (e.g. [3,4]), where the local contrast is given by the Weber fraction: |*L_i_* − *L*_mean_|/*L*_mean_ (e.g. [1]). However, both of these metrics suffer from the fact that they increase with the size of the image, which is not generally consistent with human vision [5,6]. A simple way around this is to divide these measures by the area of the stimulus. In the first case, this gives the contrast average, in the second case, if we also take the square-root, it gives a measure known as the root mean square (RMS) contrast (which is equivalent to the standard deviation of the local image values (e.g. pixels), normalized by the mean). This measure has been widely used as an image summary statistic (e.g. [1,2,7–12]) and has received some experimental support that it is relevant to human perception [2,13]; but are there other approaches that we might adopt?

One option is to consider the sort of measures that have emerged from the psychophysical investigations of contrast discrimination (also known as contrast increment detection) for sine-wave gratings (and related stimuli). Perhaps, the best known of these is that derived by Legge and Foley, where the local contrast response is given by Resp_L&F_ = *C*^2.4^/(*z* + *C*^2^), where *C* is a local measure of contrast (in per cent) and *z* is a constant. This model produces a non-saturating sigmoidal transducer for contrast, and predicts the so-called dipper function for contrast discrimination [14]. (Note that in this model and the next, the saturation constant, *z*, is important in determining the transition point in the sigmoidal contrast response function. However, when the stimuli are well above threshold (as they were here), it can be safely set to unity where its influence becomes negligible. For example, resetting this parameter to zero has a negligible effect in our toy models.) The Legge and Foley approach might be extended to the global contrast by summing the results of transduction over the entire image:

1.1 $${\text{Resp}}_{L\&F\_\mathrm{mod}}=\sum \left[\frac{C^{2.4}}{z+C^{2}}\right],$$

where Σ denotes summation over stimulus area. However, while this solution is worthy of consideration, it is a questionable candidate, since it is known to fail for global contrast discrimination, even when the potency of the area summing operation is diminished to probability summation or Minkowski summation^1^ across area [14,16,17]. For example, at moderate contrasts and above, increasing the area of a sine-wave grating has little or no effect on an observer's ability to detect small increments in contrast, whereas the modification to the Legge and Foley model above (regardless of the precise form of summation over area) predicts that performance should be better for the larger stimulus [14]. One solution to this problem might be to normalize equation (1.1) by overall stimulus area; a more effective solution was offered by Meese and Summers (M&S) in terms of their contrast gain control model [16], developed from earlier work by Foley [18]. This was devised specifically to resolve the problem above, but has also been successful in describing a wealth of other results, to which we shall return in the Discussion. The M&S gain control equation can be stated as follows:

1.2 $${\text{Resp}}_{M\&S}=\frac{\sum \left(C^{2.4}\right)}{z+\sum (C^{2})}.$$

This has a similar form to the modified Legge and Foley equation (equation (1.1)), but in equation (1.2), summation over area takes place separately on the numerator and denominator terms of the equation.

**Figure 2.**
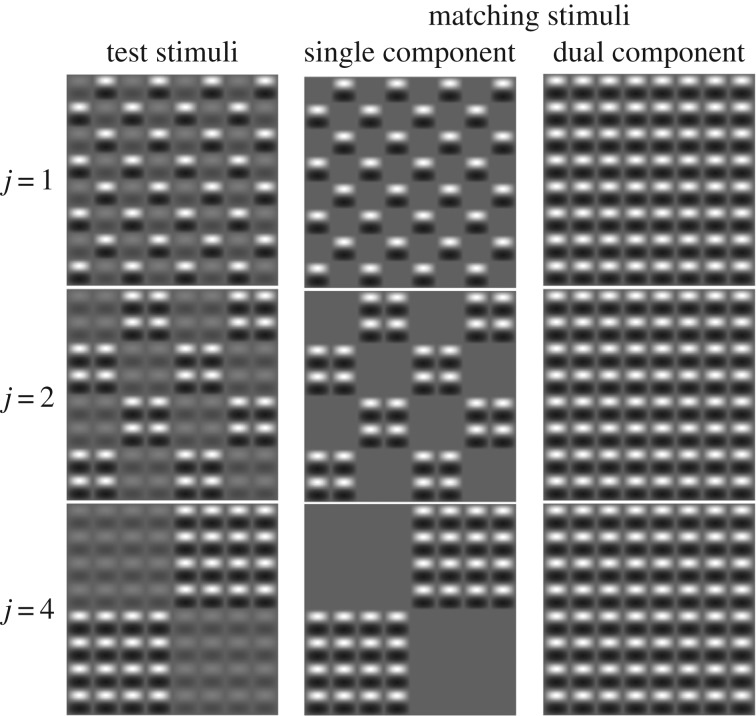
Example Battenberg stimuli (smaller than the 20 × 20 element stimuli used in the experiments). Different rows are for different cluster sizes (*j*) of elements (the *j* = 8 condition is not shown). The first column shows test stimuli; the second two columns show the two different types of matching stimuli (single and dual). In all cases, nominal ‘A’ and ‘B’ elements are interdigitated across the stimulus. In the test stimuli, these could have different contrasts (as shown here). In the single component matching stimulus, the ‘B’ contrast was set to 0%, and in the dual component matching stimulus the ‘A’ and ‘B’ contrasts were equal. Staircases were used to control the contrast of the matching stimulus to find the point of subjective equality (of global contrast) with the test stimulus. In the experiments, the ‘B’ contrast of the test stimulus was always 8%.

So how might we compare the performances of the various models and metrics above? While natural images (such as that in figure 1) are valuable for illustrating our central point, they are not well suited to testing contrast perception experimentally, since we wish to control the levels and spatial extents of local and global contrasts in a systematic way. To do this, we devised a contrast-matching task using the so-called Battenberg stimuli from a contrast detection study [19], in which the contrasts of two interdigitated regions of a test stimulus could be adjusted independently (e.g. see left column of figure 2). By matching this to the contrast of a second Battenberg stimulus, in which all of the elements had the same contrast, we assessed human perception of global image contrast and considered the method it might use by comparing the results to mathematical predictions from the competing models/metrics.

## 2. Methods

### 2.1. Apparatus and stimuli

Stimuli were presented on a Nokia Multigraph 445X monitor running at 120 Hz, using a ViSaGe system (Cambridge Research Systems, Kent, UK). The monitor was gamma corrected, and had a mean luminance of 60 cd m^−2^. At the viewing distance of 119 cm, 48 pixels subtended one degree of visual angle on the monitor. The overall stimulus width was 8.3 deg. Stimuli were presented with 14-bit greyscale resolution against a mid-grey background.

We used the ‘Battenberg’ stimuli described by Meese [19]. These were constructed from a horizontal sine-wave grating with a spatial frequency of 2.4 c deg^−1^, multiplied by a full-wave-rectified vertical sine-wave grating of half the spatial frequency. This produced a square grid of single-cycle elements (20 × 20 elements, each 20 × 20 pixels square). We adjusted the Michelson contrast of each alternate element (producing a ‘*j* = 1’ pattern), or repeating clusters of elements (producing ‘*j* > 1’ patterns), depending on the conditions of our matching experiment. Examples of 8 × 8 element samples of the stimuli are shown in figure 2.

### 2.2. Procedure

Observers viewed the monitor from a distance of 119 cm, with their head supported by a chin rest. We used a temporal two-interval contrast-matching paradigm. In one randomly assigned interval, the test stimulus was a Battenberg pattern in which half of the elements (the ‘B’ pattern) had a contrast of 8%, and the contrast of the remaining elements (the ‘A’ pattern) ranged from 0% to 32%, depending on the condition. In the other interval, the matching stimulus was another Battenberg pattern, the global contrast of which was determined by a pair of 1-up-1-down staircases [20], each of which terminated after 12 reversals. The staircases determined the contrasts of either half of the elements (with any remaining elements having 0% contrast), or all of the elements, producing our ‘single’ or ‘dual’ component matching conditions, respectively (figure 2). The stimulus duration was 100 ms and the inter-stimulus interval was 400 ms. Note that in the example stimuli in figure 2, the spatial assignment of ‘A’ and ‘B’ components is always the same (e.g. a ‘B’ element always appears in the top left of the image); in the experiments, the complementary arrangement was also used and with equal probability from trial to trial.

The observer's task was to use a two-button mouse to indicate which of the two stimuli appeared higher in overall contrast. No feedback was given for this subjective task (since there was no objective ground truth). The experiment was blocked by the contrast of the ‘A’ component, with staircases interleaved across the single and dual conditions within a block. Blocks were run in a random order, and all observers repeated the experiment four times. We fitted a cumulative log-Gaussian to the psychometric data from each repetition (consisting of the results from approximately 42 trials) to estimate the point of subjective equality (there were no lapse rate parameters). These were then averaged across repetition to give an overall estimate for each observer.

### 2.3. Observers

Three experienced psychophysical observers (the authors: T.S.M., D.H.B. and R.J.S.) completed the full experiment, performing over 10 000 trials each. All were aware of the aims of the project, and wore their normal optical correction throughout. Three naive observers also performed a limited set of experimental conditions (single- and dual-matching for the *j* = 2 condition). All three were psychophysically well practised, but none knew the details of the experiment or the models being explored. They did know that the aim of the experiment was to press buttons in order that the experimental procedure would tend towards presenting stimulus pairs that would appear to be the same or very similar in overall contrast.

## 3. Results

### 3.1. Practised observers

**Figure 3.**
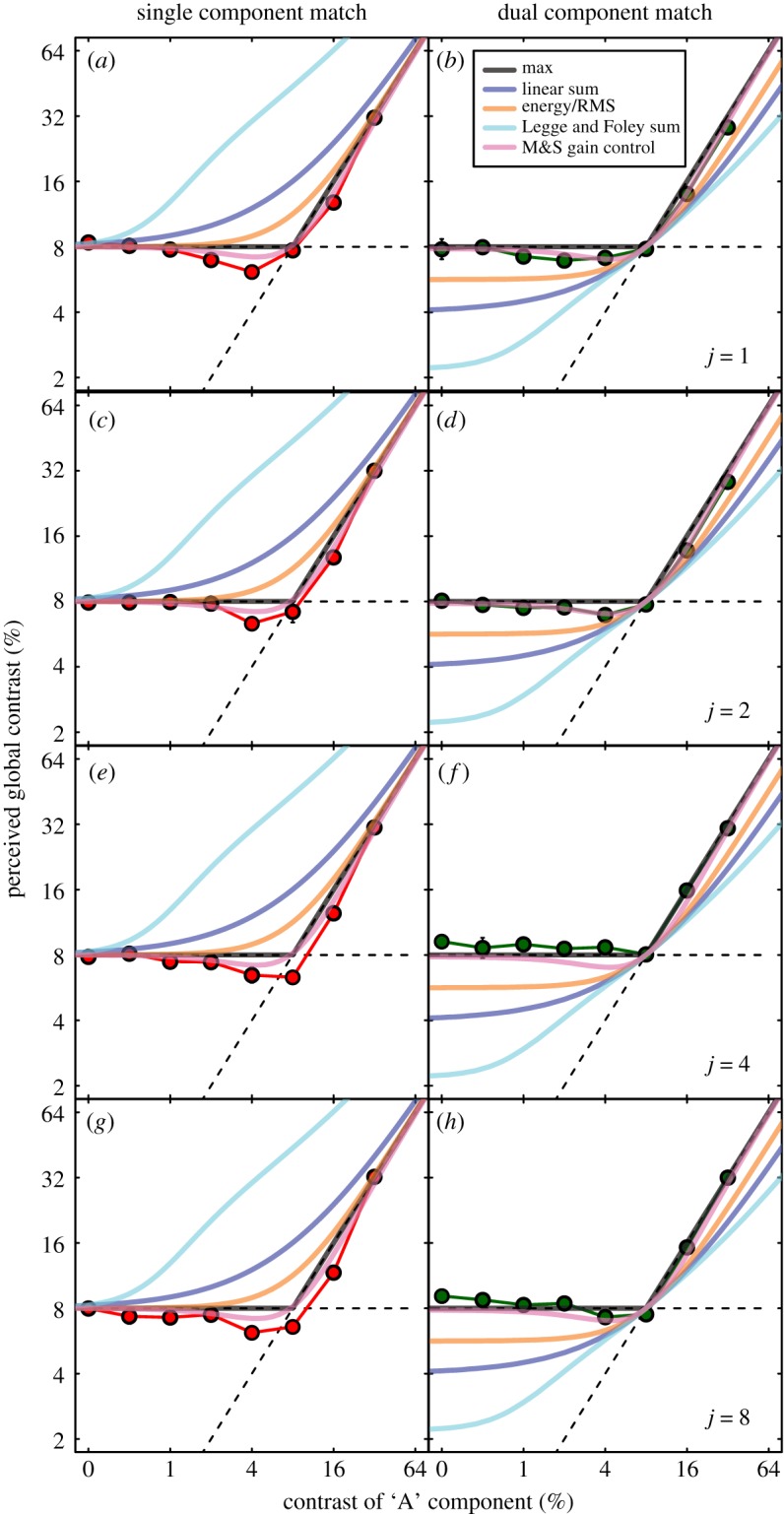
Results of the contrast-matching experiments. Results were averaged across three observers, and error bars show ±1 s.e., but in most cases are smaller than symbol size. Different columns are for the two different matching stimuli (left = single; right = dual), and different rows are for different cluster sizes (*j* = 1, 2, 4, 8, from top to bottom). The ordinate is for the contrast of the matching stimulus, and the abscissa is for the ‘A’ contrast in the test stimulus. The ‘B’ contrast was fixed at 8% and is indicated by the horizontal dashed line. The oblique dashed line has a slope of unity. The curves are model predictions (no free parameters) described in the text.

The results for the three main observers were very similar; their average is shown in figure 3. The red symbols (left column) are for the single component matches and the green symbols (right column) are for the dual component matches. The figure panels show matching contrast as a function of the ‘A’ contrast component in the test stimulus. For the lower ‘A’ contrasts, the fixed ‘B’ contrast of 8% dominated the match, whereas for the higher ‘A’ contrasts, the ‘A’ contrast dominated; the black horizontal and oblique lines indicate these two regimens, respectively. Thus, the matching functions are close to a strategy that simply picks the highest contrast in the image. However, for intermediate ‘A’ contrasts, the matching function was paradoxical: in most cases, when an ‘A’ contrast of 4% was added to the B contrast of 8%, the perceived global contrast fell below 8%. Similarly, on the right-hand side of the function, when an ‘A’ contrast of 16% was added to a ‘B’ contrast of 8%, the perceived global contrast fell below 16% (the paradoxical effects were also evident in the results for each individual observer). Thus, a MAX(*C*) operation (where *C* is the local contrast) does not explain the full matching functions. (The exceptions to the paradoxical effect were for *j* = 4 and *j* = 8 in the dual-matching condition; we shall return to this nuance in the Discussion where we offer an explanation.)

The reason for including multiple values of *j* was to assess the spatial spread of the global perception of contrast. Since the effects were so similar across *j* (particularly for the single component match), our experiments here do not point to any spatial limitations in this respect implying that, whatever the process, it extends over at least 2× (*j* = 8) elements. We have drawn similar conclusions at contrast detection threshold [16,19], though other studies suggest that the integration range might be even more extensive [15,21,22].

### 3.2. Naive observers and alternative response strategies

**Figure 4.**
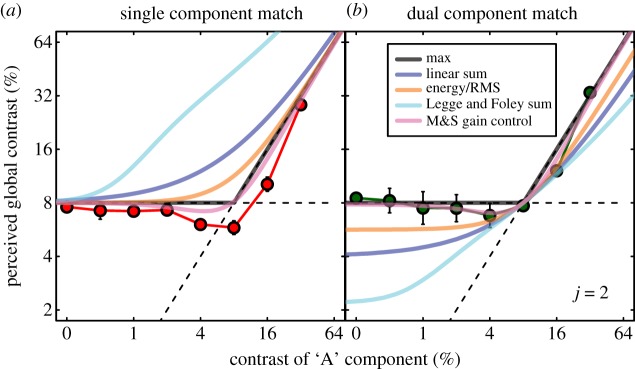
Average results for three naive observers. Details are the same as for figure 3.

During review, it was suggested to us that our practised observers might have used their expert knowledge of contrast metrics to drive the results in a particular way. We addressed this by re-running our experiment on naive observers. Their average results are shown in figure 4 for *j* = 2 and have a very similar form to those of our practised observers (figure 3). Thus, it is clear that knowledge of the experimental hypotheses is not needed to achieve this pattern of results, including the paradoxical mismatches.

Notwithstanding the result above, we wondered whether an experienced observer might be able to systematically ‘throw’ the results, should they choose to; our expectation was not high, since all of our observers were given a rapid sequence of randomly interleaved trial pairs (with two different matching stimuli, blocked across eight different ‘A’ component contrasts), with the task of deciding which image in each pair had the higher overall contrast; we found this task to be perceptual, not cognitive, whatever the details of the underlying strategy. Nonetheless, to see whether an observer could impose a non-perceptual (i.e. cognitive) strategy, D.H.B. reran the experiment adopting what he believed to be an RMS matching strategy (since our modelling in the next section suggests that visual perception of global image contrast is *not* RMS contrast). He found this very difficult (error bars were larger than normal), the matching functions were non-monotonic in a way that was not predicted by any of our models (which are described in the next section), and the results provided us with no valuable insights into the issues that we were investigating (see part 2 of the electronic supplementary material for these results). However, we should point out that with suitable training and trial-by-trial feedback, the possibility remains that it might be possible for observers to learn cognitive response strategies that would allow them to defeat the answers delivered more directly by their perceptual machinery. The extent to which perceptual behaviour might be manipulated in this way remains an open research question.

We should also emphasize that in all cases, our observers were instructed to make overall (global) contrast judgements, and this they felt able to do. However, as informal inspection of figure 1 attests, we also expect that observers would be able to make local contrast judgements. This was not the focus of our study, but raises several issues that are open to further research.

## 4. Modelling

We compared our human matching results (for the three expert observers in figure 3) to the predictions made by the various models described in the Introduction. We began by testing simple toy models that received just two image parameters: the A and B contrasts. This had the advantage of simplicity, and transparency, and was a valuable first step in developing intuitions for understanding model behaviour. Thus, the MAX() model is given by *C*_global_ = MAX(*A*,*B*) and is shown by the solid black lines in figure 3. For the stimuli here, this model also makes identical predictions to Michelson contrast (since the highest and lowest luminances in the display were always given by the element(s) with the highest contrast). Thus, although the RMS error for these models is low (1.03 dB^2^), their behaviours are not very satisfactory, since they do not predict the paradoxical effects, evident in the data. The next simplest idea was to take the linear sum (or equivalently here, the average), given by *C*_global_ = (*A* + *B*). Predictions are shown by the blue curves and are clearly inadequate (RMS error = 4.1 dB).

Next we tested the energy model, given by: *C*_global_ = (*A*^2^ + *B*^2^) and shown by the yellow curves. This improves on the linear sum (RMS error = 2.37 dB), but still fails at the knee point of the single-matching function. For the dual match results, the situation is even worse, since although the location of the knee point is correctly predicted, the upper and lower limbs of the matching function are both underestimated, quite substantially. For the stimuli here, where the test and match images were the same size, the energy and RMS models make identical predictions; hence, both of those models are inconsistent with our results. Next, we tried a version of the Legge and Foley [14] model in which we simply summed pairs of Legge and Foley sigmoidal contrast transducers to give us: *C*_global_ = *A*^2.4^/(1 + *A*^2^) + *B*^2.4^/(1 + *B*^2^). The predictions are shown by the cyan curves, and are very poor for both types of matching task (RMS error = 8.78 dB). This is the worst model that we tested, and again, because the image size was constant, normalizing by area makes no difference. However, by replacing the linear area summation operation with Minkowski summation (see footnote 1), the performance of this model improved (not shown), the predictions tending towards the MAX() model as the Minkowski exponent *k* approached infinity. For *k* = 4, the predictions are very close to the energy/RMS predictions. However, we could find no value of *k* that allowed the model to predict the paradoxical effect. Finally, we tried the M&S contrast gain control model given by: *C*_global_ = (*A*^2.4^ + *B*^2.4^)/(1 + *A*^2^ + *B*^2^). The predictions are shown by the pink curves and are clearly very good, capturing all key features of the data, including the paradoxical effect. This model also produced the best overall quantitative prediction of the results (RMS error = 0.81 dB; see appendix A for further details).

We also note that our above conclusions are supported by the modelling of results of our naive observers, where the RMS error was lower overall for the M&S model than it was for any others (RMS error = 1.23 dB; the next best was the MAX() model; RMS error = 1.61 dB).

Finally, we note that the paradoxical effect is evident in 10 out of 10 of our human matching functions (figures 2 and 3), but that of the models that we tested, this was present in only the M&S model. Thus, not only did the MAX() model lose out against the M&S model quantitatively (its RMS errors were worse), it also lost out qualitatively. Thus, it would seem that the MAX() model is missing a key ingredient that is to be found in the M&S model. Given the global nature of the psychophysical task, we suggest that this ingredient involves the global integration of the image contrast. However, the results show that not any global contrast integration metric will do, since the Legge and Foley summing model and the RMS metric, both clearly fail.

### 4.1. Further model developments

**Figure 5.**
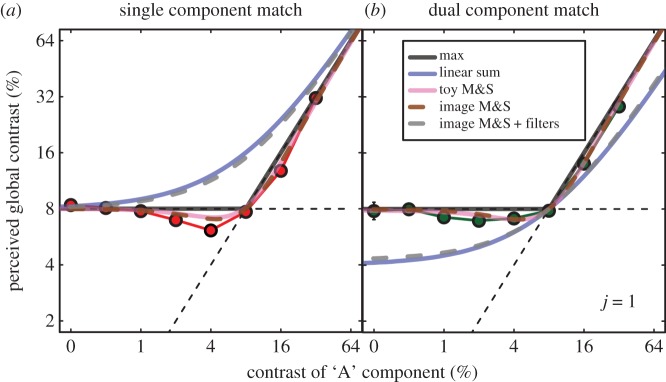
The effects of spatial filtering on the M&S contrast gain control model for *j* = 1. The blue and pink curves are replotted from figure 3. The brown curves show that applying the model pixel-by-pixel makes very similar predictions to the toy M&S model. The grey curve shows that when spatial filtering is added, the M&S model behaves much more like the unsuccessful linear sum model (blue curves).

The toy models considered above were further developed in two distinct ways. First, instead of operating on just a pair of image parameters (the ‘A’ and ‘B’ contrasts), we applied the models directly to the images, summing and maxing as appropriate on a pixel-by-pixel basis (e.g. [16,23]). Second, we added a spatial filtering stage to the front end of the models, using the same log-Gabor filter that worked very well for the contrastdetection of Battenbergs [19], and then operated over pixels (on the output of the filter) in the same way as above. (Here, we used only a cosine-phase filter, but the same results pertain with a sine-phase filter or a quadrature pair.) None of these changes helped the above failed models; but the impact on the M&S contrast gain control model was interesting. This is shown in figure 5 for the two different matching stimuli and *j* = 1. The blue and pink curves are for the toy models from before, for the linear sum and M&S models, respectively. The dashed brown curves show the effects of operating on a pixel-by-pixel basis for the M&S model, but without the spatial filtering. Not surprisingly, the predictions are very similar to those of the toy model (pink curves). The effects of spatial filtering in the M&S model are shown by the dashed grey curves: the predictions are now seriously deficient, being very similar to those for the linear sum (blue curves). The reason for this is that the footprint of the spatial filter elements (tuned to the orientation and spatial frequency of the stimulus) is larger than a single filter-element, and so the filtering is effectively performing the A + B operation of the linear sum model, which sums indiscriminately across the A/B boundaries. This interpretation was supported by our observations that as *j* increased (not shown), the filter model predictions became more like those of the toy model, because the effects of summation within the linear filter became less relevant (since the boundaries between the ‘A’ and ‘B’ regions become fewer). In other studies that measured contrast detection thresholds, the filtering was a critical feature of the model, because it was needed to account for the perfect linear summation (a factor of 2) between ‘A’ and ‘B’ contrasts when *j* = 1 [19] as well as other sub-threshold summation effects [15,22]; but in contrast-matching, we now see that the model is undermined by the inclusion of the very same filter. We will discuss the implications of this striking observation in the next section.

## 5. Discussion

To investigate the metric/process used in human spatial vision for judging the global image contrast, we performed a contrast-matching task for test stimuli containing two spatially interleaved contrast levels. Only one model provided a good account of the results, namely the M&S contrast gain control model [16]. It is gratifying that essentially the same model (see the rest of the Discussion for the caveats) has enjoyed success in several visual performance tasks including contrast detection [15,19,21,22], contrast discrimination [16,23], contrast masking [24,25], summation across eyes, orientation, space and time [17], and now also the subjective task of global contrast-matching.

### 5.1. Transducer exponents at threshold and above

One subtle difference in the model at threshold and above is the value of the transducer exponent on the numerator (i.e. the excitatory exponent). At detection threshold, this has a value of 2 [15,19,26] consistent with contrast energy detection [3,4], a strategy that suits a system in which the signals are generally unknown [27]. However, in contrast discrimination experiments, the numerator exponents are typically set to 2.4 and those on the denominator to 2.0 (e.g. [14,16]). We suspect that the true exponent is closer to 2.0 (as suggested by the threshold work), and that the extra 0.4 that is needed to fit the dipper region of contrast discrimination functions actually derives from uncertainty, which can masquerade as a nonlinear transducer [15,28,29]. If this were correct, then the level of intrinsic uncertainty would need to be fairly low (to raise the effective model exponent from 2.0 to the slightly higher value of 2.4). In fact, accumulating evidence from threshold work suggests that intrinsic uncertainty is indeed quite low [15,26,30]. The M&S model here used exponents of 2.4 and 2.0 to be consistent with previous suprathreshold work, but re-running the model with a numerator exponent of 2 and a denominator exponent of 1.6 (the denominator exponent must be lower to avoid response saturation) had a negligible effect on its predictions.

### 5.2. The paradoxical effect and contrast–contrast

In the paradoxical effect, the presence of one contrast signal causes the perceived overall contrast to decrease. In some respects, this is similar to the phenomenon known as contrast–contrast, where the surround contrast causes a decrease in the perceived contrast of a central target [31]. However, there are important differences between the two effects: in contrast–contrast, there is a repulsive effect between two contrasts, whereas here, the effect is attractive: the addition of a low contrast causes a higher contrast to appear lower. The tasks are also different: in contrast–contrast, judgements are made on a central local contrast in the presence of a surround that is irrelevant to the task, whereas here, observers were tasked with a contrast judgement of the entire image. In other work [32], instances have been found where surround contrast can cause a reduction in central contrast, even when the surround contrast is lower than in the centre. However, this surround suppression was found to increase, as the surround contrast increased. If this were the sole explanation of our paradoxical effect, we should expect it to be even stronger in the single match (red curves) when the ‘A’ contrast increased from 4% to 8%, the opposite of what we often found (e.g. see figure 5*a*). Furthermore, a formal attempt to save this idea using a MAX() operator in conjunction with suppression was found to fail badly (see part 3 of the electronic supplementary material). Overall then, we conclude that the paradoxical effect here is not the same phenomenon as contrast–contrast or simple surround suppression, though their causes might well be related, involving contrast interactions between the centre and surround [24,33,34]. Indeed, recent electroencephalography work by Baker & Wade [35] has found direct evidence for the substantial contrast suppression between ‘A’ and ‘B’ components for the Battenberg stimuli used here (their figure 4*a*,*b*); they also found some evidence for the paradoxical effect (their figure 3*a*).

It is difficult to provide a transparent intuitive account for why the M&S model predicts the paradoxical effect, but its origins are the same as those found in paradoxical binocular contrast-matching [36,37] and also paradoxical psychometric functions for dichoptic masking, spatial masking, temporal masking and orientation masking [25]. In the latter experiments, 2IFC performance actually falls below chance (i.e. d’ becomes negative) as the test contrast *increases* (over a limited range of the psychometric function). This aspect of the model also relates to a performance-task phenomenon known as *dilution masking*, in which the target contrast becomes inappropriately summed with the irrelevant image contrast carried by other visual mechanisms that have been suppressed by the target [16,17,25]. In short, these effects happen in the model, because the benefits of integration are outweighed by the detrimental effects of suppression over a critical range of signal contrasts (see Baker *et al.* [25] for further details).

### 5.3. Key model failures and a recommendation

Our rejection of the linear sum (and average) models is perhaps not surprising, since this measure receives little or no support from the human vision literature beyond short-range summation of contrast within V1-like receptive fields [19]. Likewise, and for the reasons outlined in the Introduction, we were not surprised to find that our results rejected the Michelson contrast metric (though this remains a potentially valuable parameter for local contrast, of course) and the MAX() operation (e.g. see our discussion of figure 1*a*,*c* in the Introduction). Rather more important are the rejections of: (i) the extension to the Legge and Foley model, and (ii) the RMS model. Elaborated versions of the former (e.g. [18,38]) have been attractive to other vision researchers when models have been extended to natural images, particularly when the summation process is set to Minkowski summation (e.g. [39,40,41]), presumably because of their success with gratings and the like [14,18,38,40]. However, this model is known to fail in contrast discrimination tasks involving summation over area [14,16], and its failure here (figure 3) suggests that using it for reasons of expedience might well lead to errors. The RMS metric has good intuitive appeal in terms of image description [39,42,43], but because it fails for contrast discrimination of gratings (masking levels do not increase with pedestal contrast when additive internal noise is assumed) and now also for contrast-matching (where the assumptions about internal noise are less important), we suggest that if this metric is to be used in a human context, then this should be done with care. On the other hand, since the application of equation (1.2) is just as easily applied to an image (e.g. using MATLAB) as the calculation of RMS contrast, then if the psychophysics, neurophysiology and image-processing communities wish their summary statistic of global image contrast to relate to human perception, we advise that they use equation (1.2), or similar (e.g. see the section on transducer exponents above) instead.

### 5.4. Implications of dual component matching for *j* = 4 and *j* = 8

As we mentioned earlier, the results for the practised observers for *j* = 4 and *j* = 8 in the dual-matching condition are a little different from the others. It is as though they have been slid upwards by a small amount, abolishing the paradoxical effect and raising the left hand limb slightly above the MAX() prediction. The perceptual implication of this slippage of the left most points is that the dual-matching stimulus (right column in figure 2) is seen to be slightly lower in contrast than the single-matching stimulus (middle column in figure 2) when they have the same Michelson contrast (i.e. every element has a contrast of 8%). (This is also implied by the two data points for *j* = 4 and 8 in the single-matching condition (left column in figure 2) when the ‘A’ contrast was 8% (fifth data point from the left), which is the same physical stimulus pair that we have just described, but with the target and match swapped around.) Why might this mismatch occur? Let us suppose that the excitatory integration region (numerator) were in the order of 4 elements across, but the inhibitory integration region (denominator) were spatially more extensive (consistent with explanations of surround suppression [33] and contrast masking [24]). Then for *j* = 4, excitation would be the same for both stimuli, but inhibition would be less for the single stimulus because of the large gaps in the surround region. This would mean that the overall response to the single stimulus would exceed that to the dual stimulus, and so its contrast would look slightly higher, just as we found (and described above). On the other hand, the smaller patches in the *j* = 1 and *j* = 2 stimuli mean that the excitation and inhibition caused by the single stimulus would each be half of that caused by the dual stimulus, and hence the overall responses would be the same. Since the slippage effect is also evident for *j* = 8, the implication is that either the excitatory integration region was adjusted to match the cluster size for each of these stimuli, or that it is fixed in size, somewhere between the two (e.g. 6 elements). However, since these experimental effects were very small, and since they were also evident in our naive observers at *j* = 2 (figure 4), suggesting individual differences (i.e. different ranges of *j* over which observers show the discrepant result), we saw little merit in trying to pursue the details of this in the modelling here, but the issue might draw more focused experimental consideration in the future.

### 5.5. Spatial filter synthesis?

The detrimental effects of spatial filtering in our model were not anticipated, particularly since this is such an important part of our understanding of threshold vision [15,19]. So, how can it be that the filters appear to simply vanish in our suprathreshold matching task? This aspect of our work warrants further investigation, but it is tantalizing to suppose that the apparent lack of filtering might be the consequence of a process of filter synthesis; the argument works as follows. At contrast detection threshold, there is no sensory evidence for the precise details of the target and so the system applies the best hard-wired basis filter that it has at its disposal [19] in an attempt to optimize the signal-to-noise ratio; because its footprint is larger than a single image element, this necessarily blurs the pattern boundaries of our *j* = 1 stimuli. However, once the stimulus is well above threshold—as in the experiments here—then there is good visual evidence for the form of the stimulus, and so the visual system reforms its front end by appropriately weighting its basis filters (e.g. [44–47]). One approach might be to construct a convolution kernel that is, or approximates, a non-oriented delta function from the weighted sum of the oriented basis filters (or wavelets), and then sum across those responses (following squaring and gain control) to derive the global image contrast. Such an approach might be quite straightforward in principle, since, unlike for steerable filters (e.g. [45]), the filter weights needed for this application would be fixed (i.e. precomputed and not image dependent). Our experiment and analyses do not provide direct evidence for this idea, but our observations are as follows: (i) our results imply that the spatial filtering becomes transparent above threshold, (ii) the visual system is not in any sense able to switch out the basis filters at the front end of the cortical visual stream, and (iii) by constructing a filter with a delta impulse response in the stimulus region, image blurring (by a single narrow-band basis filter) is abolished. This last point would explain why neural blurring [19] and filling in [16] affect perceptual judgements at threshold, but not above, where the gaps in our Battenbergs are readily seen and are not filled in (figure 2). A scheme such as the one we propose would hand over the role of the individual basis filter-elements to other suprathreshold tasks, such as perceptual grouping and segmentation across and within local regions of analysis (e.g.[48,49]). This would not rule out the possibility, of course, that individual filter-elements might still be picked out to optimize the signal-to-noise ratio in suprathreshold performance tasks such as masking (e.g. [18]). On the other hand, other work involving suprathreshold tasks, such as stereo matching and motion detection in pink noise, concluded that oriented narrowband spatial filters cannot be picked out for these tasks [50].

### 5.6. Outstanding issues

Several other issues remain outstanding, chiefly those concerning image segmentation. For example, while the high contrast patch (Z) in figure 1*c* is readily segmented (perceptually) from the rest of the TV image, it remains to be seen whether it must also be excluded from the global measure of contrast in the model. Indeed, this raises the thorny questions of: (i) what determines the boundaries for regional assessment of image contrast—just how global is *global*?—and (ii) how many such (nested) assessments can be made across a single image. Whether an analysis based on spatial frequency sub-bands, as suggested by Peli [1], might be used, remains to be seen.

## 6. Conclusion

Here, we have extended our previous work on contrast detection and contrast discrimination to contrast-matching. We find that all of these perceptual tasks can be understood within a single framework of contrast integration over space though, intriguingly, spatial filter characteristics are transformed from what is found at contrast detection threshold to what is effectively a transparent process (for the task here), above threshold. We suggest that this might be because the visual system synthesizes a filter with an impulse function that is a delta function for suprathreshold contrast perception, thereby preserving the perceptual image, protecting it from filter blur and other neural distortions. Finally, not only does our framework serve as a model of human contrast perception but it can also serve as an image contrast metric for image processing applications: it is as straightforward to compute as the popular RMS contrast metric, but has the distinct advantage that it is more closely related to human visual perception.

## Supplementary Material

Extra image analysis, experiment and model

## Footnotes

1 In Minkowski summation, each of the terms to be summed is first raised to a power *k*, then the overall result is raised to the power 1/*k*. Thus, when *k *= 1, we have the linear summation of terms in equation (1.1). The power has been set to *k* = 4 in traditional models of Minkowski summation, though see [15].

2 Contrast in dB is given by 20 log_10_(*C*).

## Data accessibility

The raw experimental data are available at: https://doi.org/10.6084/m9.figshare.1595854.v3.

## Appendix A: Detailed results of model analysis

Here, we provide a more detailed presentation of the performances of the various models that we considered against our two datasets (the average of three experts/authors and the average of three naive observers). Note that for no model/metric was there any parameter fitting, since where parameters were arguably a part of the model (the Legge and Foley model and the M&S model), their values were set according to the traditional values used elsewhere and as described in the main body of the report. The RMS error (in dB) is shown for each model and each dataset and *j* value in table 1 (see table caption for details). The entries highlighted in red are the lowest values (best models) in each column. In nearly every case, this is the M&S contrast gain control model—the only one to capture the paradoxical matching effect (see main body of report). The MAX() model typically came second, but in a few instances outperformed the M&S model. This was for the dual-matching condition when *j* = 4 or 8. However, as we noted in the main body of the report, these data were a little unusual, in that they appeared to be slid vertically a little, relative to the others, implying a mismatch in perceived contrasts across the single and dual configurations when all stimulus elements had a contrast of 8%. This nuance (discussed in the main body) is not a feature of our modelling, but was compensated in our analysis here by sliding each dataset to intersect the *y*-axis at a contrast of 8%. With this manipulation (middle block in table 1), the M&S model once again won out against the MAX() model. The reason for this is that every one of our datasets showed the paradoxical matching effect that was a property of only the M&S model, but without the normalization procedure, the data rode high in some conditions, putting them slightly closer to the MAX() model.

**Table 1.** RMS errors (in dB) for the various model predictions (different rows) for the average results of the three expert observers (top two blocks) and the three naive observers (bottom block). (The green and red blocks are for the single and dual component matches, respectively, in figures 3 and 4. The purple blocks show the errors combined across the two types of match (green and red), and the mauve blocks show the errors combined across different values of *j*. In the blocks in middle row, each dataset was slid vertically to normalize the intercept with the *y*-axis at 8% (18 dB). The RMS errors highlighted in red are the lowest in each column. For comparison, the average standard errors (across observers) for the practised observers were 1.1 dB (for each of *j* = 1, 2, 4, 8) and for the naive observers was also 1.1 dB (cf. the purple blocks).)

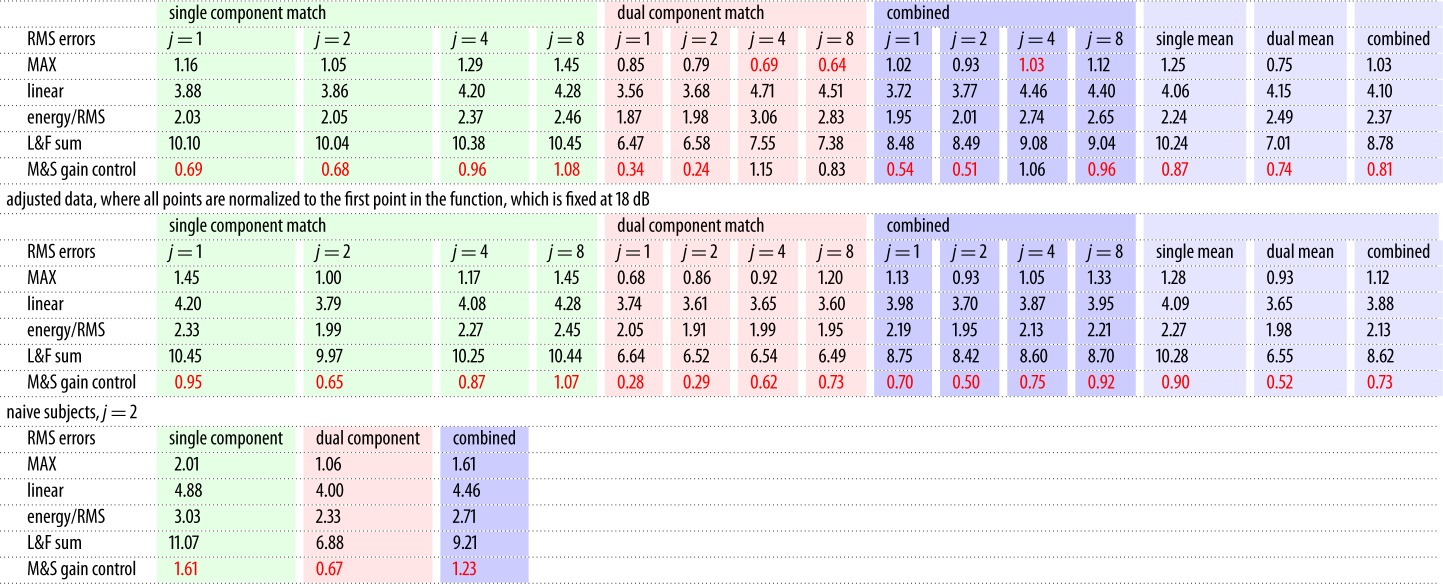

Note that in the main body of the paper, we reported the RMS errors from the rightmost columns in the top and bottom blocks of table 1.
