# Supplementary material for: Perception of global image contrast involves transparent spatial filtering and the integration and suppression of local contrasts (not RMS contrast)
Source: R Soc Open Sci. 2017 Sep 6;4(9):170285. doi: 10.1098/rsos.170285 (PMC5627075; doi:10.1098/rsos.170285)
Supplement: Extra image analysis, experiment and model [file rsos170285supp1.docx]

**Supplementary material to Meese, Baker and Summers (2017):** *Perception of Global Image Contrast Involves Transparent Spatial Filtering and the Integration and Suppression of Local Contrasts (not RMS contrast)*

Open Science.

**Part 1: Pink noise image analysis**

We wanted to make a formal comparison of local image contrasts across pairs of static achromatic images. To do this, we created an image of pink noise (1/*f* filtered noise, where *f* is spatial frequency). The image was 512 pixels square. We copied this image (Image 1) to a second image (Image 2) and scaled the global contrast of Image 2 in factor increments of 0.05 (from zero to one). We compared the local contrasts of Image 1 with the local contrasts of Image 2 in each of two ways (to be described), and plotted the proportion of comparisons in which Image 1 had the higher local contrast as a function of the contrast factor of the second image. The results are shown in Fig S1.

The green curve is for when we used Weber contrast (see Introduction of main paper), as a measure of local contrast. Thus, for each image pair, we compared each local measure of contrast from one image on a pixel by pixel basis with each local measure of contrast in the other image, also on a pixel by pixel basis (i.e. there were 512^2^x512^2^ local comparisons per contrast factor). When the global image contrast of Image 2 was zero, the local comparisons always favoured the image with the higher global contrast. However, as the contrast of the second image increased, the proportion of comparisons that favoured the first (higher contrast) image decreased. For example, by the time the second image had half the global contrast of the first (an easily discriminable difference), 29% of the local comparisons picked the wrong image (compared to 50% by chance when the two images had the same global contrast; far right).

The blue curve is for when we used RMS contrast (see Introduction of main paper), as a local measure. We did this for each non-overlapping 32x32 pixel sub-region of Image 1 with each 32x32 pixel sub-regions of Image 2 (i.e. there were 16^2^x16^2^ local comparisons per contrast factor). By the time that the global contrast of the second image was 90% that of the first image, 27% of the local comparisons picked the wrong image. Image contrast differences of around this magnitude (a 10% difference in relative contrast) are discriminable for gratings (e.g. Legge & Foley, 1980) and achromatic faces (unpublished observations).

Figure S1. Comparisons of two different measures of local contrasts of pink noise samples as a function of the global contrast of the second image proportional to the first. See text for details.

We repeated the analyses shown in Fig S1 with several different original noise samples, and we also made comparisons across image pairs where the noise samples were different across the pair. The results were indistinguishable from those shown here. The point of this analysis is that a local comparison of image contrast is a poor proxy for making a (more) global comparison of image contrasts, particularly if the difference in global image contrasts is not that large (e.g. only 10%). This provides general motivation for asking our research question: how might the visual system do this?

**Part 2: Additional experiment**


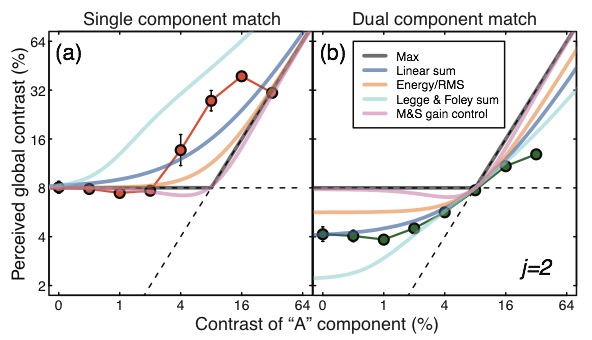


Figure S2. Results for a single practiced observer (DHB, one of the authors) for a supplementary experiment in which he adopted a cognitive strategy of trying to make a contrast match based on RMS contrast. Error bars show ±1 SE. The model curves are as for those in the main body of the report.

The results of our supplementary experiment are shown in Fig S2 for DHB. The methods were the same as those used in the main experiment (e.g. the results are the averages of four replications), but instead of trying to make a contrast match based on direct perceptual experience, the observer contrived the match using a cognitive strategy based on his best guess of what RMS contrast might be (a measure that our main experiment suggests is not the one used by human observers). In fact, his strategy would seem to be closer to the linear sum (blue curve) than RMS (yellow curve), but none of the models does a particular good job of describing these data. Whether this observer, or others, might be trained to match their data more successfully to particular model curves remains unclear.

**Part 3: Additional model**

Here we report the results of an additional model (Max + suppression) given by:

*Resp* = MAX(*A*^2.4^, *B*^2.4^)/(*z*+*A*^2^+*B*^2^), (Eq S1)

where *z* = 1, and *A* and *B* are the component ‘A’ and ‘B’ contrasts in percent. This can be thought of as the MAX() model, plus self and surround suppression and exponentiation or, equivalently, the M&S model, with the linear summation on the numerator replaced by the MAX() operator. The model prediction (no free parameters) is shown by the brown curve in Figure S3 (the other curves and the data are identical to those in Figure 3 of the main report). It’s combined RMS error was 9.17dB, making this the worst model that we examined. The crux of the problem for this model is where one of the stimuli contains twice as many stimulus elements of the same or similar contrast as the other one (e.g. the point at which ‘A’ contrast = 8% in the single component match condition). In this situation, the level of suppression (the denominator of the model equation) is twice as great for the stimulus with the greater number of non-zero contrast elements (ignoring the small contribution from *z*). In the M&S model (pink curve) this heavy suppression is offset by the benefits of the linear combination of stimulus contrast on the numerator of the model equation (e.g. Eq. 2 in the main report). However, for the MAX() + suppression model, this does not happen; the MAX() operator returns the *same* image contrast to the model numerator for both stimulus configurations. Manipulating the values of the exponents in this model (e.g. *p* = 1.4, *q* = 1) produced some quantitative changes to model performance, but did not improve on its qualitative behaviour. Since a large body of evidence points to the involvement of surround suppression in early luminance contrast vision, and specifically for these stimuli (Baker & Wade, 2017), we conclude that a signal selection strategy (i.e. MAXing) is not being used by observers in making the contrast match in our experiments.


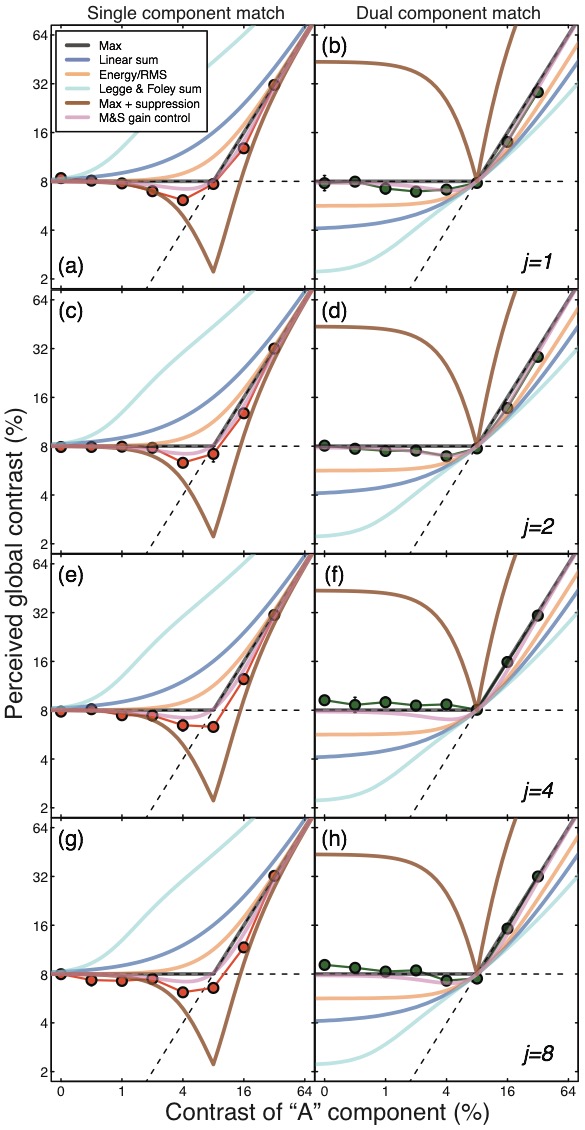


Figure S3. This is identical to Figure 3 in the main body of the report with the addition of a further model prediction (brown curve) described in the text.

**References**

Baker, D. H. & Wade, A. R. (2017) Evidence for an optimal algorithm underlying signal combination in human visual cortex. *Cerebral Cortex*, **27**, 254-264.

Legge, G.E. & Foley, J.M. (1980) Contrast masking in human vision. *Journal of the Optical Society of America*. **70**, 1458–1471.
